# Identification of Loci Through Genome-Wide Association Studies to Improve Tolerance to Sulfur Deficiency in Rice

**DOI:** 10.3389/fpls.2019.01668

**Published:** 2020-01-15

**Authors:** Juan Pariasca-Tanaka, Cedric Baertschi, Matthias Wissuwa

**Affiliations:** Crop, Livestock and Environment Division, Japan International Research Center for Agricultural Sciences (JIRCAS), Tsukuba, Japan

**Keywords:** rice, sulfur, deficiency tolerance, quantitative trait locus, genome-wide association studies, *aus*

## Abstract

Sulfur (S) is an essential nutrient for plant growth and development; however, S supply for crop production is decreasing due to reduced inputs from atmospheric deposition and reduced application of S-containing fertilizers. Sulfur deficiency in soil is therefore becoming a widespread cause of reduced grain yield and quality in rice (*Oryza sativa* L). We therefore assessed the genotypic variation for tolerance to S deficiency in rice and identified loci associated with improved tolerance. Plants were grown in nutrient solution with either low (0.01 mM) or high (1.0 mM) supply of S. Plants grown under low-S treatment showed a reduction in total biomass, mainly due to a marked reduction in shoot biomass, while root biomass and root-to-shoot ratio increased, relative to plants under high-S treatment. Genome-wide association studies (GWAS) identified loci associated with root length (*qSUE2-3, qSUE4,* and *qSUE9*), and root (*qSUE1, qSUE2-1, and qSUE3-1 and qSUE3-2*) or total dry matter (*qSUE2, qSUE3-1,* and *qSUE11*). Candidate genes identified at associated loci coded for enzymes involved in secondary S metabolic pathways (sulfotransferases), wherein the sulfated compounds play several roles in plant responses to abiotic stress; cell wall metabolism including wall loosening and modification (carbohydrate hydrolases: beta-glucosidase and beta-gluconase) important for root growth; and cell detoxification (glutathione S-transferase). This study confirmed the existence of genetic variation conferring tolerance to S deficiency among traditional aus rice varieties. The advantageous haplotypes identified could be exploited through marker assisted breeding to improve tolerance to S-deficiency in modern cultivars in order to achieve sustainable crop production and food security.

## Introduction

Sulfur (S) is an essential element for growth and development in living organisms; however, animal digestion, including that of humans, is unable to metabolize sulfur directly. Therefore, animals depend on the consumption of plant biomass for the supply of organic S, such as sulfur-containing amino acids (methionine and cysteine), vitamins (thiamine and biotin), and many other secondary compounds (Maruyama-Nakashita et al., 2003; Lunde et al., 2008; Kopriva et al., 2015).

Sulfur-containing amino acids are found in very small amounts in legumes, while most cereals contain high levels for example rice (*O. sativa L*.) contains 190% of the reference value, egg protein (Mariotti, 2017). Rice is a staple food for more than half of the world, providing more than 50% of the caloric supply for low-income persons. A reduction in rice yield and grain quality, due to inadequate amount of S supply, could therefore have a negative impact on the world's food security.

In plants, S is also an essential macronutrient needed in similar amounts to Phosphorus for growth and development. Apart from being a vital component of amino acids and vitamins, S is also a component of coenzymes, cofactors, lipoic acid, glutathione, sufloquinovosyl diacylglycerol, and many other secondary compounds (Suzuki, 1978; Lunde et al, 2008; Kopriva et al., 2015).

Sulfur is abundant in nature as sulfate (SO_4_^2-^); however, to utilize this abundant sulfate, plants must invest energy for its reduction. Plants can incorporate sulfate by both primary and secondary sulfate assimilation pathways. In the primary pathway, S is taken up as sulfate, then reduced to sulfite (SO_3_^2-^), sulfide (S^2-^), and then incorporated into the amino acid skeleton of O-acetylserine to form cysteine. Subsequently, methionine and other compounds containing reduced forms of S are produced. In the secondary pathway, sulfate is used as a donor for the sulfation reaction, for synthesis of larger compound such as brasssinosteroids, sulfoflavonoids, and glucosinolates (Kopriva et al., 2015).

Symptoms of S deficiency in plants during vegetative stages includes initial chlorosis of young leaves, which spread to the entire plant, reduced tillering, and stunted growth. Symptoms of S deficiency are similar to that of N deficiency. Yellowing of older leaves is seen in N-deficient plants, while yellowing of young leaves occurs in S-deficient plants, since S does not move readily in the plant (Chandel et al., 2003).

Sulfur deficiency in soil was rare in the past; however, it has become increasingly inadequate in recent history, due to factors like increased removal of nutrients by intensive cropping (estimated at 1.8 kg S t^-1^ grain) (Dobberman and Fairhurst, 2000), reduced application of S-containing N and P fertilizers (ammonium sulfate and single superphosphate) (Yoshida and Chaudhry, 1979), and reduced atmospheric inputs (from reduced industrial pollution). Furthermore, strong solar irradiation increases the S requirement of plants, which could further exacerbate the effect of S deficiency in soil (Resurrection et al., 2001). Therefore, in the last 2 decades, reports of S deficiency in crops has increased worldwide (Scherer, 2001), including some Asian countries like China, India, Indonesia, Philippines, Sri Lanka, and Thailand (Blair et al, 1978). Similarly, the problem of S deficiency in crops has also been reported in many African countries (Yamaguchi, 1999; Tsujimoto et al., 2013).

Although the advantage of fertilizer application to alleviate S deficiency has been widely reported (Blair et al., 1980), the applicability of this practice by low-resource farmers is uncertain. Therefore, the identification and selection of rice genetic resources with enhanced tolerance to S-deficiency in soil, and improved S utilization efficiency would be very desirable to attain sustainable rice production and food security.

Recently, genome-wide association studies (GWAS) based on single nucleotide polymorphism (SNP) has emerged as an alternative method to study association mapping using genetically diverse rice populations. GWAS can determine the strength of the association between a genotype and phenotype and identify loci, genes and alleles that are associated with speciﬁc traits. In rice, GWAS has been successfully applied to dissect the genetic bases of several traits (Zhao et al., 2011), including seedling vigor (Wang et al., 2018), root morphology (Biscarini et al, 2016), flowering time (Zhao et al, 2011), yield and grain size (Feng et al., 2016; McCouch et al., 2016), as well as ozone and aluminum tolerance (Famoso et al, 2011; Ueda et al., 2015), phosphorus-utilization efficiency (Wissuwa et al., 2015), and root efficiency (Mori et al., 2016).

The rice *aus* group is a minor sub-population which originated from Bangladesh and India (Klush, 1997; Norton et al., 2018). It harbors novel loci that, confer tolerance to abiotic stress (Famoso et al., 2011; Lee et al., 2018), and enhance root development (Mori et al, 2016). Therefore, the *aus* group would be an excellent genetic reserve in the search for new sources of tolerance to S deficiency in rice.

Despite the growing problem of S deficiency in soil and its negative effects on crop yield and food security, the genetic aspect of S deficiency tolerance in rice is not yet well understood. We therefore aimed to use GWAS to identify candidate loci, conferring tolerance to S deficiency in an *aus* rice panel. We hypothesize that such association mapping will identify several loci associated to tolerance, which could be used for the improvement of sensitive modern cultivars, to attain sustainable crop production and food security of an important staple food, rice.

## Materials and Methods

Three experiments were conducted as part of this study. Since nutrient solutions typically contain large amounts of S as a companion-ion for major nutrients (K, Mg, Zn), a low-S nutrient solution was tested in experiment 1. This was followed by screening a rice GWAS panel (experiment 2) and a validation trial with contrasting genotypes (experiment 3).

## Experiment 1

Seeds of different genotypes (IR64, DJ123, Nerica4, Tsipala, WAB56-104) were sown and grown in modified Yoshida nutrient solution (using de-ionized water) under different S-treatments: no-S (0-S), low-S (0.01 mM S) or high-S (1.0 mM S). The full-strength Yoshida solution (1X) is composed of: N, 2.86 mM (as NH_4_NO_3_); P, 0.05 mM; K, 1mM; Ca, 1mM; Mg, 1mM; Mn, 9 μM; Mo, 0.5 μM; B, 18.5 μM; Cu, 0.16 μM; Fe, 36 μM; Zn, 0.15 μM (modified from Yoshida et al., 1971). To obtain the low-S solution, all sources of S were substituted as follows: KCl for K_2_SO_4_, MgC1_2_ for MgSO_4_, ZnC1_2_ for ZnSO_4,_ and CuCl_2_ for CuSO_4_ (**Supplementary Table S1 in Data Sheet 1**). A stock solution of 10 mM MgSO_4_ was used as S source for the low-S treatment. The experiment was set in a factorial (Sulfur treatment and genotype, 2 × 5) arrangement in a randomized complete block design (RCDB), with three replications.

## Experiment 2

### Plant Material and Growth Condition

A GWAS panel of 98 accessions belonging to the *aus* sub-species of rice was selected from the High-Density Rice Array (HDRA; McCouch et al., 2016). De-hulled seeds were obtained from the International Rice Research Institute (IRRI, Philippines), and multiplied at a JIRCAS experimental field on Ishigaki Island, Japan. Seeds from each genotype were disinfected using 1% bleach solution, germinated in petri dishes at 30°C for 2 days, transferred to a floating mesh, and grown for 8 days in a solution containing 0.1 mM Ca (CaCl_2_) and 0.012 mM Fe (Fe-EDTA). Subsequently, plants were grown either under low-S or high-S conditions, as described above.

In screening for the ability of rice plants to efficiently utilize a limited amount of S, we employed a similar approach to that used by Rose et al. (2011) in screening for P utilization efficiency (PUE). If all accessions are grown individually in a container with a known amount of the limiting nutrient, and if it is assured that this amount was fully taken up, then the biomass produced can be directly used to estimate nutrient use efficiency as follows: biomass (g) per nutrient applied (mg). Thus, for the low-S treatment 10-day old plants of each accession were transferred to 1-L black bottles (two plants per bottle). Bottles contained 0.3× Yoshida solution with 0.01 mM S (total 0.32 mg S available for uptake by 2 plants; as for Experiment 1). Plants were grown for 10 days in these bottles and during this period it was expected that they deplete the low amount of S in the solution. This was confirmed by drawing a 10 mL aliquot from the bottles and analyzing its S concentration using ICP-AES (inductively coupled plasma optical-emission spectroscopy). Sulfur concentrations in aliquots were similar to that of the blank, confirming complete S uptake (data not shown). Having ensured that S was fully taken up, plants were transferred to a 200-L container and grown in a no-S Yoshida solution until harvest (30 days after sowing, DAS).

For the high-S treatment (control), the 10-day old plants were directly transferred and grown in a 200 L container containing high-S solution (1.0 mM S), until harvest. In general, the pH was frequently monitored and adjusted to a range of 5.7 - 5.9. A flowchart of the experiment is shown in **Supplementary Figure S1 in Presentation 1**.

The experiment was conducted in a controlled environment green house at JIRCAS, Tsukuba, Japan. Growth conditions were as follows: temperature fluctuated from 28 to 33°C day time and around 25°C during night time, and relative humidity ranged from 30-50%. Plants were exposed to natural light during the entire growth period. The experiment was performed in a factorial (Sulfur treatment and genotype, 2 × 98) arrangement in a randomized complete block design (RCDB), with three replications (block) carried out in consecutive periods from March to Aug of 2017.Parameters evaluated during harvests were: plant height, number of leaves (green and dead leaves), number of tillers, root length, and root and shoot dry matter.

### Sulfur Determination

Sulfur concentration in aliquots of nutrient solution was determined using inductively coupled plasma atomic-emission spectrometry, (ICP-AES; ICPE-9000, Shimadzu, Japan) using an S standard solution (ICP grade, Wako, Japan) to prepare the standard curve. This measurement was performed to confirm the complete uptake of S in the low-S treatment. Three independent replications were taken per each sample (total number of samples: 5)

### Association Mapping

For the phenotype dataset, the overall performance of all rice accessions for each trait was calculated as the best linear unbiased prediction (BLUP, Henderson, 1975) using the R software (R Core Team., 2013), with the following simple mixed model:

$$y_{ij}=\mu +g_{i}+\;r_{j}+e_{ij},$$

in which *y_ij_* refers to phenotype, *µ* overall mean, *g_i_*: accession effect considered as random, *r_j_*: replicate effect considered as fixed, and *e_ij_*: residual effect considered as random.

The association analysis was then performed using: a) the resulting phenotypic BLUP values; b) the HDRA genotypic dataset (McCouch et al., 2016) composed of 700 K SNPs evenly distributed across the rice genome; and, c) the software Trait Analysis by aSSociation, Evolution and Linkage 5.0 (TASSEL, Bradbury et al., 2007).

The genotype dataset was filtered as follows: heterozygotes were set as missing values, minor allele frequency (MAF) was set to 0.03, and minimum count to 105 (0.82). The resulting dataset after curation was then used for association mapping using mixed linear model procedure (MLM), 3 principal components (PCA) and a kinship matrix. The associated loci were determined based on significant threshold of –log(10)(p) >5 (arbitrary threshold), and peaks having at least three consecutive SNP (above the threshold) as described by Wang et al. (2018). The marker effects at the significant position were extracted from the output of the MLM procedure for both alleles.

### Linkage Disequilibrium and Haplotype Analysis

Linkage disequilibrium (LD) analysis to define LD blocks surrounding the significant SNPs was performed by Haploview 4.2 (Barret et al., 2005), using confidence intervals (Gabriel et al., 2002).

### Selection of Putative Candidate Genes

Gene models and their respective annotations were obtained from the Rice Annotation Project Database (RAP-DB, https://rapdb.dna.affrc.go.jp/) for each significant peak and their surrounding linkage block. However, genes annotated as ‘(retro)transposon', ‘hypothetical' or ‘unknown' were excluded from the analysis. Putative candidate genes were then selected based on their annotated function and gene ontology (http://www.geneontology.org/). Gene expression pattern was obtained from the Rice XPro database (http://ricexpro.dna.affrc.go.jp/). Sequence for the genotype DJ123 was obtained from the Schatz Lab-Johns Hopkins University (http://schatzlab.cshl.edu/data/rice/).

### Statistical Analysis

The effects of sulfur treatment, genotype and their interaction on different traits were estimated using a two-way ANOVA, and multiple comparisons were performed using Tukey's honest significant difference (HSD) *post hoc* test (Statistix 9.0 Software). Box plots were generated by R software (R Core Team, 2013).

## Experiment 3

This experiment was performed to confirm the phenotypic effects identified in experiment 2, and to investigate the expression pattern of selected candidate genes. Seeds of a selected set of contrasting genotypes were grown and evaluated as described in experiment 2. The experiment was conducted with a factorial (Sulfur treatment and genotype, 2 × 2) arrangement in a randomized complete block design (RCDB) with four replications.

The selected contrasting genotypes were divided into two groups: a) Santhi Sufaid 207, Kangro, Juma and DJ123; and b) Surja Mukhi, Harbhoondi, Khadasiya3, and Andikulan. The first group harbors the favorable haplotypes for total dry matter. The Asian mega variety IR54 was included as reference.

### Tissue Sampling, RNA Extraction and PCR Conditions

At harvest (30 das), four independent biological replications for each sample were quickly taken, flash-frozen in liquid nitrogen, and stored at -70°C until analysis. Total RNA was extracted using the RNeasy Plant Mini Kit (Qiagen), following the manufacturer instruction manual. Total RNA (around 400 ng) was then reverse transcribed (RT) using the PrimeScript RT Enzyme Mix I (Takara, Japan). RT-PCR was performed using RT (first strand-cDNA) as template, gene-specific primers, and Taq polymerase enzyme (Takara, Japan).

Quantitative PCR (qPCR) was performed using 1 ng RT template and SYBR Premix ExTaq (Perfect Real Time, Takara, Japan), using the Mini Opticon Real-Time PCR system (BioRad, USA) as previously described by Pariasca-Tanaka et al. (2009). Serial dilutions of RT product were used to determine the efficiency of each primer. A set of rice genes including Elongation factor (ELF-1), Glyceraldehyde 3-phosphate dehydrogenase (GAPDH) and Ubiquitin (Ubi) was used as internal controls. Relative expression levels between samples were calculated using the delta–delta comparison and expressed as fold changes. The normalized data were analyzed by ANOVA. The list of primers used in this study is shown in **Supplementary Table S2 in Data Sheet 1**.

## Results

### Experiment 1

Plants were grown in a modified Yoshida solution where all sources of S were substituted (refer to material and method) to test the response of a set of rice cultivars to S-deficiency treatments.

**Figure 1:**
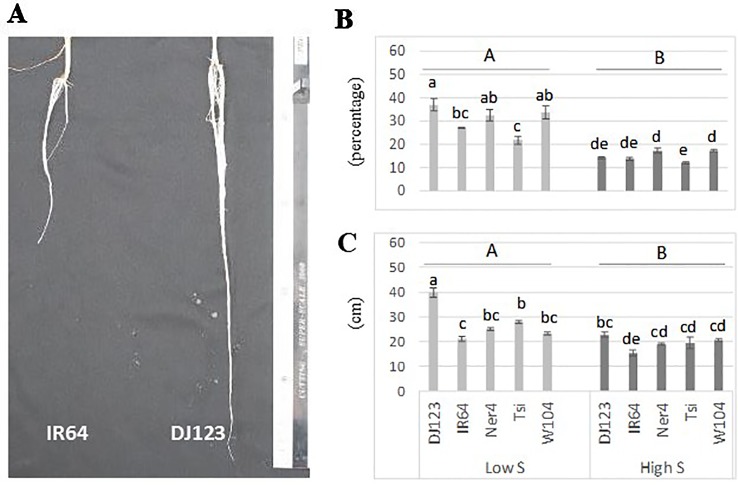
Root elongation **(A)**, root:shoot ratio **(B)** and root length **(C)** of rice plants grown under low-S and high-S treatment. Data represent the mean (± standard error, SE) of three independent replications (n=3) and analyzed by two-way ANOVA. Different letters indicate significant difference within either factor: S treatment (capital letter) or genotype (small letter) according to Tukey's Honest Significant Difference test (*P* < 0.05). ANOVA results are provided in **Supplementary Table S4-1 in Data Sheet 1**. Cultivars, DJ123; IR64; Ner4, NERICA4; Tsi, Tsipala; W104, WAB56-104.

Plants grown without any supply of S (0-S) showed a very limited growth, and their leaves spots rapidly turned from chlorotic to necrotic (**Supplementary Figure S2 in Presentation 1**). In contrast, plants grown with 1 mM S (high S) showed normal growth while those grown in low-S treatment (0.01 mM) showed slow growth and their leaves exhibited the characteristic symptoms of deficiency for this nutrient (interveinal chlorotic spots). In addition, plants grown in low-S condition showed significant root elongation and increased root:shoot ratio when compared to those grown under high-S treatment (**Figures 1A**-**C**). Among the studied genotypes, DJ123 belonging to the *aus* sub-species, showed better adaption to low-S conditions when compared to modern cultivars IR64 and Nerica 4.

### Experiment 2

Since DJ123 was the best performer in experiment 1, the *aus* panel was chosen for screening to identify loci associated with tolerance to S deficiency. Plants were grown in either low S (0.01 mM) or high S (1 mM) treatment. Result showed that low-S treatment caused a significant reduction in shoot and total biomass, where in the larger effect was the significant increase in root length and root biomass (**Table 1**). The initial characteristic chlorotic symptoms lead to an increase in the number of dead leaves. Moreover, significant reduction in plant height and number of leaves and tillers was also observed (**Table 1**).

**Table 1:** Summary of two-way analysis of variance (ANOVA) from phenotypic traits measured in plants grown under low-S and high-S treatment.

	Plant height (Ht)	Maximum root length (RLmax)	Number of tillers (Till)	Number of leaves (Lvs)	Number of dead leaves (deadL)	Root dry matter (RDM)	Shoot dry matter (ShDM)	Total dry matter (TotDM)	Root/shoot ratio (r/sh)
Genotype (G)	***	***	***	***	***	***	***	***	***
Sulfur treatment (S)	***	***	***	***	***	***	***	***	***
LS	61.9	52.5	1.5	9.0	3.2	290.1	456.4	746.5	66
HS	82.1	24.9	2.3	11.4	1.5	141.6	618.1	759.7	23.3
GxS	***	***	*	***	***	*	***	***	ns
n	364	364	364	364	364	364	364	364	364
Mean	71.9	38.7	1.9	10.2	2.4	214.8	533.5	748.3	44.6
SE	0.7	0.7	0.1	0.1	0.1	5.5	10.3	12.8	1.2
Min	34	13	1	1	1	47	123.5	193.5	13.2
Max	106	69	4	31	6	541	1313.5	1552	95.83

ns, non-significant (P > 0.05), *significant (P ≤ 0.05), **(P ≤ 0.01), ***(P ≤ 0.01). LS, Low-S; HS, high-S treatment.

**Figure 2:**
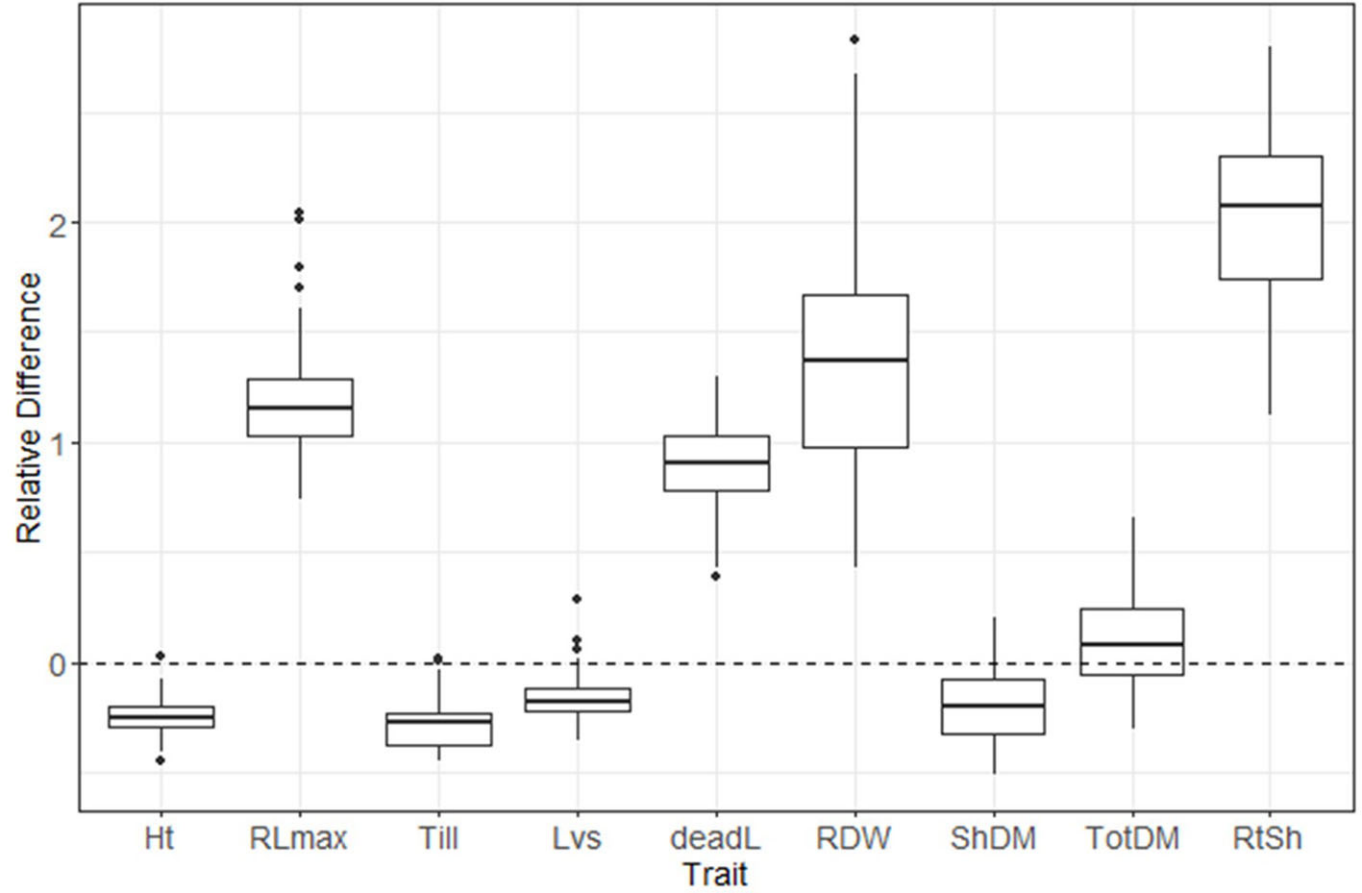
Distribution of relative difference ([low S - high S]/high S) values from evaluated phenotypic parameters. Thick lines inside the box represent the median of the distribution, while the lower and upper boundaries represent the first and third quartile, respectively. Relative difference was calculated as: (low-S minus high-S)/high-S. Ht, plant height; RLmax, maximum root length; Till, number of tillers; Lvs, number of leaves; deadL, number of dead leaves; RDM, root dry matter; ShDM, shoot dry matter; totDM, total dry matter; RtSh, root:shoot ratio.

A box plot shows the phenotypic variation in each evaluated trait as affected by S treatment (**Figure 2**). The relative difference between treatments ([low-S value minus high-S value]/[high-S value]) was largest for root:shoot ratio and root biomass, followed by root length and number of dead leaves (high-S condition), while number of leaves showed the least change between treatments. Shoot biomass, plant height and number of tillers values were higher in high-S condition.

A correlation analysis between phenotypic traits indicated that despite the increase in root:shoot ratio, the total dry matter remained most closely correlated with shoot dry matter, and moderately correlated with plant height and number of leaves, and weakly correlated with dead leaves (**Table 2**). Root dry matter and root length were moderately correlated, despite that both significantly increased under S-deficiency condition.

**Table 2:** Correlation coefficient between phenotypic traits measured in plants grown in low-S treatment.

	Ht	RLmax	Till	Lvs	deadL	RDM	ShDM	TotDM
Plant height (Ht)								
Maximum root	**0.288**							
length (RLmax)	**							
Number of tillers	**0.419**	**0.169**						
(Till)	***	*						
Number of leaves	**0.420**	**0.125**	**0.786**					
(Lvs)	***	*ns*	***					
Number of dead	**-0.049**	**0.127**	**0.180**	**0.370**				
leaves (deadL)	*ns*	*ns*	*ns*	***				
Root dry matter	**0.659**	**0.285**	**0.359**	**0.337**	**-0.155**			
(RDM)	***	**	***	***	*ns*			
Shoot dry matter	**0.800**	**0.208**	**0.592**	**0.566**	**-0.078**	**0.836**		
(ShDM)	***	*	***	***	*ns*	***		
Total dry matter	**0.778**	**0.244**	**0.528**	**0.503**	**-0.112**	**0.932**	**0.978**	
(TotDM)	***	*	***	***	*ns*	***	***	
Root/shoot ratio	**-0.461**	**-0.051**	**-0.510**	**-0.512**	**-0.171**	**0.032**	**-0.486**	**-0.308**
(r/sh)	***	*ns*	***	***	*	*ns*	***	**

ns, non-significant (P > 0.05), *significant (P ≤ 0.05), **(P ≤ 0.01), ***(P ≤ 0.01).

### Association Mapping

The genotyping dataset (from HDRA 700 K SNPs) and the phenotypic BLUP values were used for association mapping in each S treatment. For low-S treatment, a Mixed Linear Model (MLM) identified several quantitative trait loci (QTLs) associated with root length in chromosome 2 (*qSUE2-3*), 4 (*qSUE4*) and 9 (*qSUE9*); root dry matter in chromosome 1 (*qSUE1)*, 2 (*qSUE2-1*) and 3 (*qSUE3-1 and qSUE3-2)*; and total dry matter in chromosome 2 (*qSUE2-2*), 3 (*qSUE3-1*), 6 (*qSUE6*), and 11 (*qSUE11*) (**Table 3**). Moreover, for the high-S (control) treatment several QTLs were identified; though in different chromosomes compared to low-S treatment. The QTLs were found in chromosome 4 and 6 for root length; in chromosome 7 and 12 for root dry matter; and in chromosome 2 and 7 for total dry matter (**Table 3**).

**Table 3:** Quantitative trait loci (QTL) associated to root length, root, and total dry matter in GWAS using a mixed linear model (MLM).

Trait		Loci name	Chr	SNP denomination	SNP position (bp)	P	Minor allele	
							^1^MAF	^2^Effect
**Root length**								
	**LS**	***SUE2-3***	2	SNP-2.21491708	21,497,578	3.40E-06	0.17	5.4
		***SUE4***	4	SNP-4.32776574	32,961,688	5.39E-06	0.35	2.4
		***SUE9***	9	SNP-9.15018718	15,019,720	3.63E-07	0.34	5.2
	**HS**		4	SNP-4.24473685	24,658,824	7.62E-06	0.47	3.05
			6	SNP-6.21637246	21,638,244	7.38E-06	0.06	5.29
**Root Dry Matter**								
	**LS**	***SUE1***	1	SNP-1.12111994	12,113,021	6.20E-06	0.14	49.1
		***SUE2-1***	2	SNP-2.4411478	4,411,482	9.23E-06	0.41	79.8
		***SUE3-1***	3	SNP-3.19346088	19,347,578	2.32E-07	0.10	54.3
		***SUE3-2***	3	SNP-3.28010215	28,017,162	6.38E-06	0.41	76.3
	**HS**	***SUE7***	7	SNP-7.22383573	22,384,567	4.75E-06	0.08	32.9
		***SUE12***	12	SNP-12.22908597	22,942,139	9.63E-06	0.05	46.7
**Total Dry Matter**								
	**LS**	***SUE2-2***	2	SNP-2.16027627	16,033,498	1.40E-06	0.08	335.4
		***SUE3-1***	3	SNP-3.19346088	19,347,578	2.75E-06	0.10	299.1
		***SUE6***	6	SNP-6.292614	293,615	5.35E-06	0.06	442.8
		***SUE11***	11	SNP-11.17486608	17,952,751	7.06E-06	0.04	480.1
	**HS**		2	SNP-2.22318649	22,324,519	6.90E-06	0.28	181.8
			7	SNP-7.7306443	7,307,439	2.94E-06	0.05	325.9

^1^MAF, minor allele frequency. ^2^Marker effect.

The minor allele frequency and effect of alleles were also determined in each locus (**Table 3**). Minor alleles exhibited favorable effects on root dry matter in *qSUE1,* and *qSUE3-1*. Likewise, for total dry matter the minor alleles had higher effect in *qSUE2-2, qSUE3-1, qSUE6,* and *qSUE11*. In the opposite, for root length (*qSUE2)* the minor allele had a small effect compared to the major allele.

**Figure 3:**
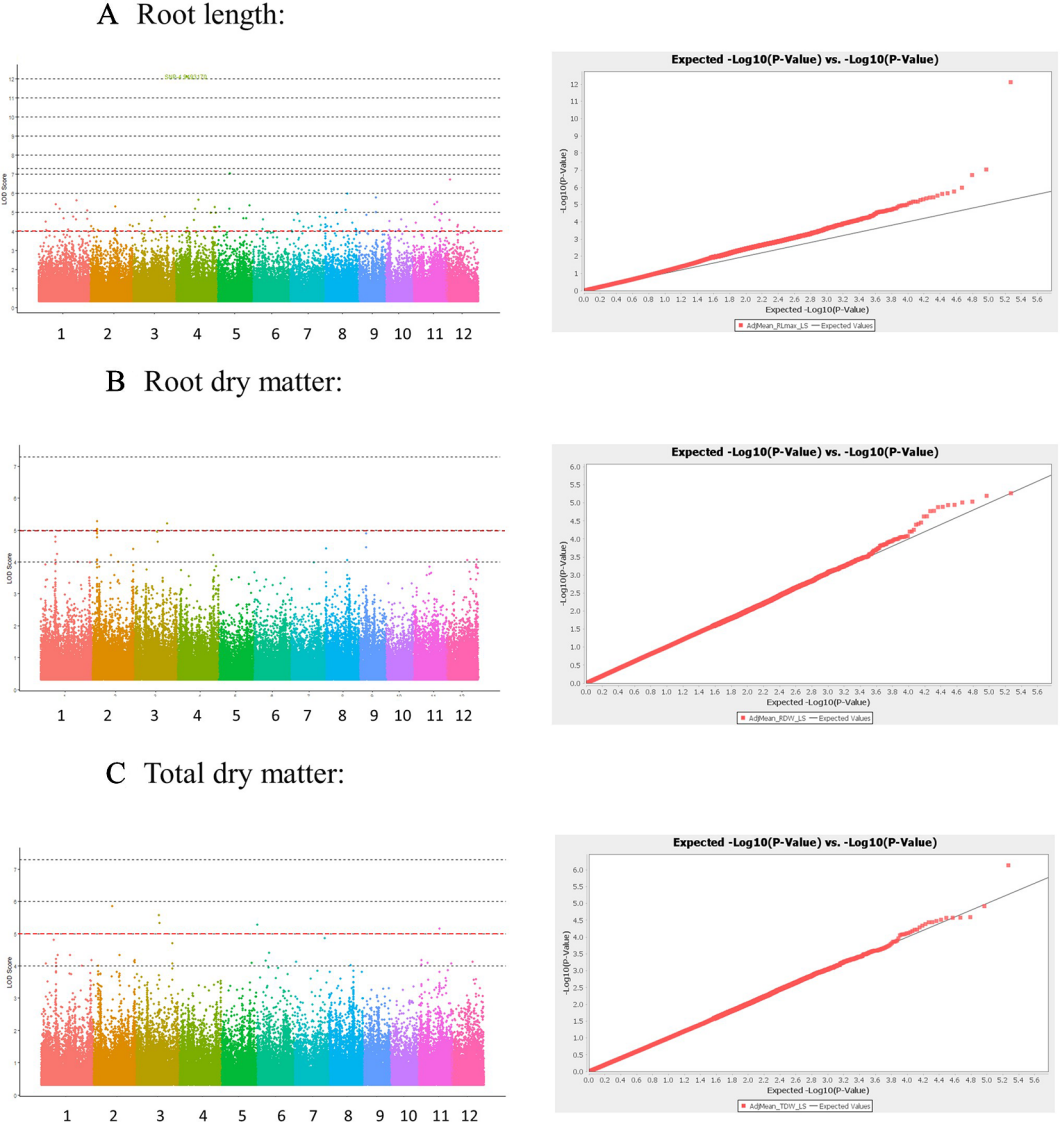
Manhattan and Quantile-quantile (QQ) plots derived from GWAS analysis for traits: **(A)** Root length, **(B)** Root dry matter, and **(C)** total dry matter. Manhattan plot shows negative logarithmic ((-log10 (*P*)) values of association for each SNP (Y axis), and SNP location along the 12 chromosomes (colored bar in X axis). Red line indicates a -log10 (P value) threshold of 5. QQ plots X shows the expected -log10 (p value) on X-axis, and measured -log10 (p value) on Y-axis.

The Manhattan plots and QQ plots for each associated QTL are shown in **Figure 3** (Low-S treatment) and **Supplementary Figure S3**-**1 in Presentation 1** (High-S treatment). A complementary GWAS analysis was performed using the phenotypic ratio of low S/high S result showed that several QTLs were consistently found in both analysis approaches (low-S and ratio Low/high S) (**Supplementary Figure S3**-**2 in Presentation 1**).

### Selection of Putative Candidate Genes

The LD block for several significant loci is shown in **Supplementary Figure S4 in Presentation 1**. LD block was clearly delineated for most peaks, except for that of *qSUE3*. Gene models contained in the LD block were retrieved from RAP-DB, and those annotated as unknown and hypothetical proteins were removed. Putative candidate genes were therefore selected based on their functional annotation and GO molecular function (**Table 4**, and **Supplementary Table S3 in Data Sheet 1**).

**Table 4:** List of candidate genes in each QTL.

RAPdb	MSU (LOC)	Annotation	Reference
**Root length - *qSUE2*-3**		
Os02g0564000	Os02g35590	Similar to glutathione S-transferase.	Kumar and Trivedi, 2018
Os02g0564400	Os02g35630	Similar to CLPX (Clp protease regulatory subunit X) 3B ATPase.	
Os02g0564700	Os02g35660	Similar to BHLH transcription factor.	Hudson and Hudson, 2015
**qSUE4**			
Os04g0647300	Os04g55360	Similar to H0811D08.10 protein.	
Os04g0647900	Os04g55420	Leucine-rich repeat 2C N-terminal domain containing protein	
***qSUE9***			
Os09g0418000	Os09g25090	Similar to CBL-interacting protein kinase 16.	
Os09g0419500	Os09g25190	Similar to ubiquitin-protein ligase/zinc ion binding protein.	Shu and Yang, 2017
**Root dry matter - *qSUE1***			
Os01g0318700	Os01g21610	Similar to ABC1 protein (Fragment).	
Os01g0319000	Os01g21630	Similar to pectin acetylesterase	
Os01g0318000	Os01g21580	Similar to esterase/lipase/thioesterase family protein.	
***qSUE2-1***			
Os04g0164900		Similar to starch debranching enzyme	
Os04g0165600	Os04g08340.1	Peptidase S26A signal peptidase I family protein	
***qSUE3*-1**			
Os03g0448700	Os03g33590	Interferon-related developmental regulator domain containing protein.	
Os03g0556600	Os03g35600	Serine/threonine protein kinase-related domain containing protein.	Diédhiou et al., 2009
***qSUE3-2***			
Os03g0700700	Os03g49380	Similar to lipoxygenase	
**Total dry matter - *qSUE2-2***			
Os02g0471500	Os02g27220	Protein phosphatase 2C domain containing protein	
Os02g0472700	Os02g27310	Similar to receptor-like serine-threonine protein kinase	
***qSUE6***			
Os06g0104300	Os06g01490	Similar to pectinesterase-like protein	
Os06g0104200	Os06g01480.1	Similar to OsNAC7 protein	
***qSUE11***			
Os11g0503900	Os11g30810	Sulfotransferase family protein.	Chen et al., 2012
Os11g0505300	Os11g30910	Sulfotransferase 2C resistance to rice stripe virus	

Candidates genes for root length, locus (*qSUE2*), would be glutathione S-transferase (Os02g0564000), the BHLH transcription factor (Os02g0564700), and the PTO kinase interactor (Os02g0565500). In the case of *qSUE4* the candidate is a *protein containing Leucine rich repeat domain (Os04g0647900),* while for *qSUE9*, candidate genes are ubiquitin conjugating enzyme binding group (Os09g0419500) and CBL-interacting protein kinase 16 (Os09g0418000).

For root dry matter, the candidates for locus *qSUE1-1* would be genes encoding pectin acetylesterase (Os01g0319000) and ABC1 protein (Os01g0318700). Starch debranching enzyme (Os02g0164900) and peptidase S26A protein (Os02g0165600) are candidates for *qSUE2-1,* while genes encoding interferon-related developmental regulator (Os03g0448700) and serine/threonine protein kinase (Os03g0556600) are candidates for *qSUE3*-1. For *qSUE3*-2, the candidate encodes for lipoxigenases (Os03g0700700)

For total dry matter, candidates for *qSUE2-2* are a protein phosphatase (Os02g0471500) and protein kinase (Os02g0472700), while a pectinesterase protein (Os06g0104300) for *qSUE6.* For locus *qSUE11*, there were two genes encoding sulfotransferase (Os11g0503900 and Os11g0505300) flanking the LD block (**Table 4**).

### Experiment 3

**Figure 4:**
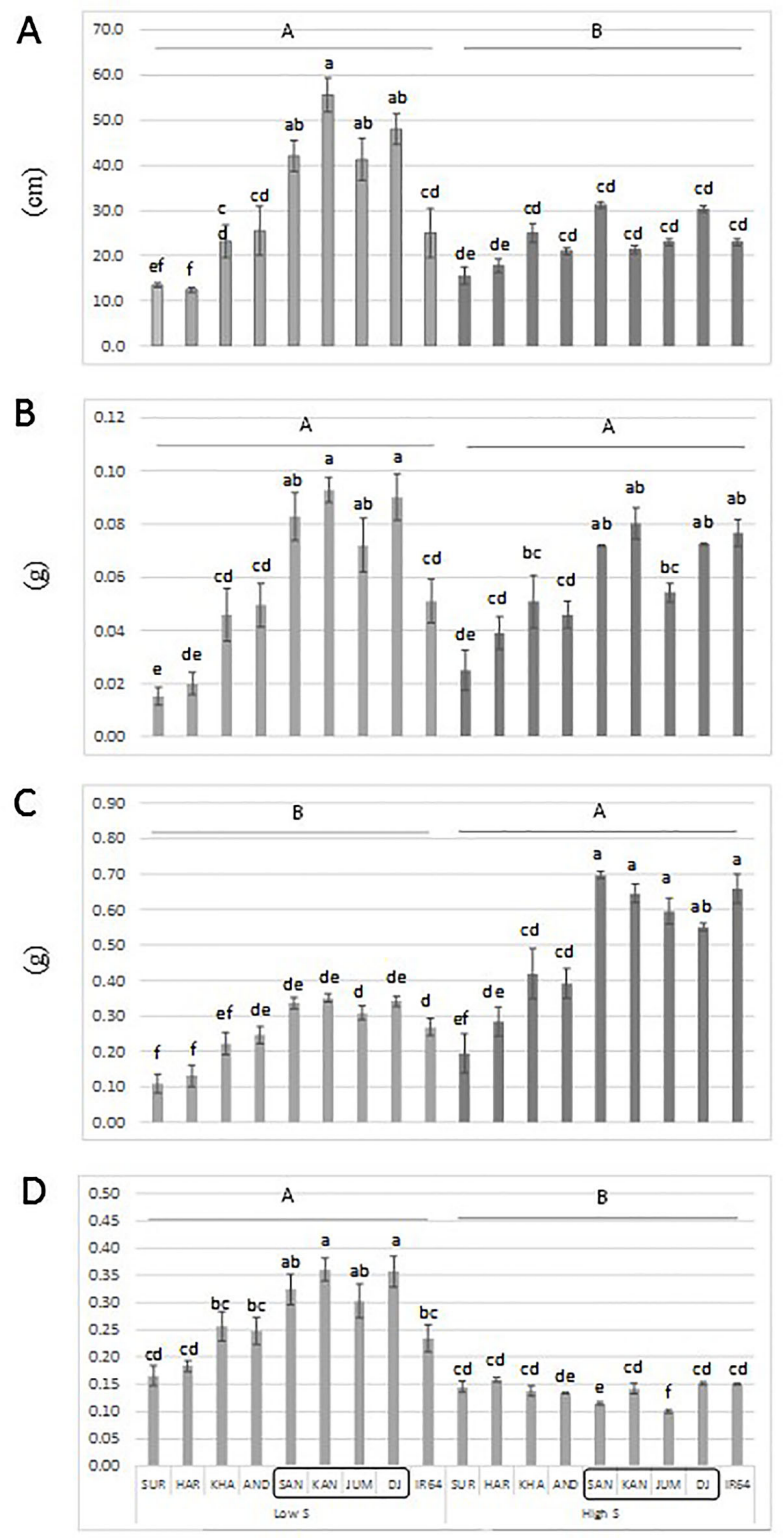
Root length **(A)**, root dry matter **(B)**, total dry matter **(C)** and root:shoot ratio **(D)** of a set of *aus* genotypes grown under low-S or high-S condition. Genotypes included in the box harbor the favorable haplotype for total dry matter. Data represent the mean (± standard error, SE) of three independent replications (n = 3) and analyzed by two-way ANOVA. Different letters indicate significant difference within either factor: S treatment (capital letter) or genotype (small letter) according to Tukey's Honest Significant Difference test (*P * < 0.05). ANOVA results are provided in **Supplementary Table S4-2 in Data Sheet 1**. SUR, Surja Mukhi; HAR, Harbhoondi; KHA, Khadasiya3; CHU, Chungur Bali; AND, Andikulan; NP, NP 125; SAN, Santhi Sufaid207; KAN, Kangro; JUM, Juma; NCS, NCS 160; DJ, DJ123.

An additional experiment was carried out to confirm the performance of a set of genotypes with contrasting haplotypes associated with total dry matter. The genotypes: Santhi Sufaid 207, Kangro, Juma and DJ123, which harbor the favorable haplotypes for root and total dry matter, showed a significant increase in root length, root and total dry matter, and root:shoot ratio compared to the unfavorable haplotype group: Surja Mukhi, Harbhoondi, Khadasiya3, and Andikulan. The Asian mega variety IR64 was included as reference (although its haplotype was not determined) (**Figure 4** and **Supplementary Table S4 in Data Sheet 1**, ANOVA). A complementary analysis using relative values for low S/high S is presented in **Supplementary Figure S5 in Presentation 1**.

### Gene Expression

The *qSUE11* is surrounded by two genes: *qSUE11-1*, Os11g0503900 (cds: 987 nuc, amino acids: 328), and *qSUE11-2*, Os11g0505300 (cds: 1188 nuc, amino acids: 395). Both genes encode the sulfotransferase family protein since they contain the Sulfotransfer_1 domain, PF00685 (Hirschmann et al., 2014). An alignment comparison between both genes showed 55.2 and 62% similarity at the nucleotide and amino acid sequence, respectively (**Supplementary Figure S6**-**1 in Presentation 1**). The gene Os11g0503900 was partially sequenced in Surja Mukhi and Santhi Sufaid 207 (harboring the favourable- and unfavorable haplotype, respectively). Result showed no difference in their nucleotide alignment (**Supplementary Figure S6**-**2 in Presentation 1**). In addition, a BLAST search for both genes against the genome sequence of accession DJ123 (Schatz Lab-Johns Hopkins University, Schatz et al., 2014) showed the presence of SNPs and indel for both cases: Os11g0503900 (98.6% similarity, 3 indel and 16 SNP) and Os11g0505300 (98.7% similarity, with single 6 indel, and 2 short indel) (**Supplementary Figures S6**-**3**, **S6**-**4 in Presentation 1**).

**Figure 5:**
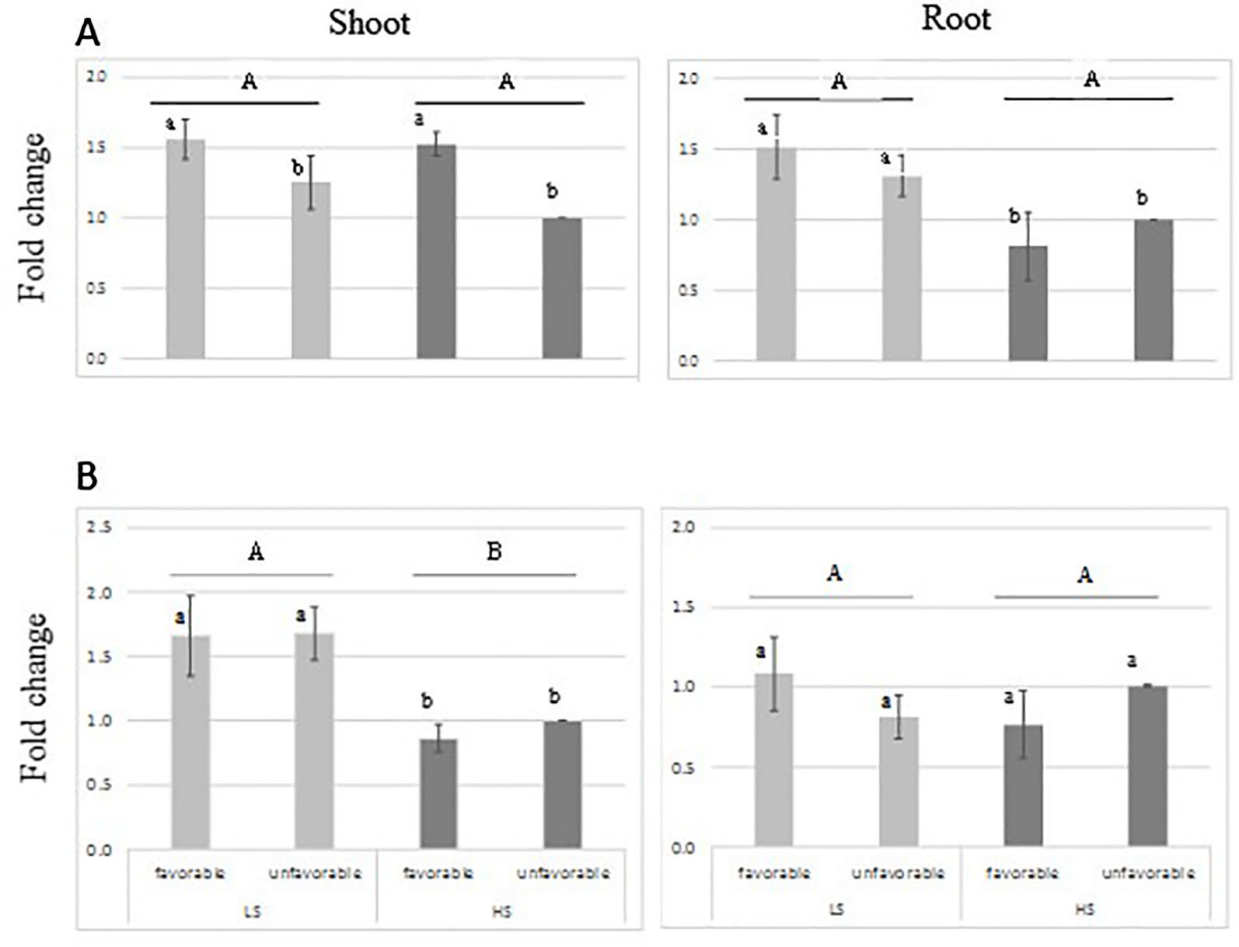
Relative expression of sulfotransferase genes: Os11g0503900 **(A)** and Os11g0505300 **(B)** in root and leaves tissue from genotypes harboring favorable/unfavorable haplotypes for total dry matter. Plants were grown either under low-S or high-S condition. Data represent the mean (± standard error, SE) of three independent replications (n=4) and analyzed by two-way ANOVA. Different letters indicate significant difference within either factor: S treatment (capital letter) or haplotype group (small letter) according to Tukey's Honest Significant Difference test (*P* < 0.05). ANOVA results are provided in **Supplementary Table S4**-**3 in Data Sheet 1**. Favorable haplotype group: Santhi Sufaid 207, Kangro, Juma and DJ123. Unfavorable group: Surja Mukhi, Harbhoondi, Khadasiya3 and Andikulan.

RT-qPCR analysis indicated that the transcript-level abundance of both sulfotransferase genes Os11g0503900 and Os11g0505300 was consistently low (**Figure 5**), as it was reported by Chen et al. (2012) and Rice X-Pro. The transcript level of both genes was not induced by S treatment; however, higher transcript level was found for Os11g0503900 in shoot tissue of the favorable-haplotype group. ANOVA result is presented in **Supplementary Table S4 in Data Sheet 1**.

## Discussion

To date, most work related to S deficiency has focused on Arabidopsis; this study thus aimed to elucidate the genetic aspect of tolerance to S deficiency in rice, an agronomically important cereal crop model. Our initial experiment (Experiment 1) testing a modified Yoshida solution, with all sources of S substituted (**Supplementary Table S1 in Data Sheet 1**) indicated that the low-S supply (0.01 mM) was enough to cause measurable symptoms in 30-day-old plants, which could be used to screen and identify plants that are tolerant to S-deficiency.

In the same experiment, the genotype DJ123 (belonging to the *aus* sub-species) showed better performance compared to modern rice cultivars (IR64 and Nerica 4), indicating that the *aus* group could be a good target for further screening. The next step of our study, therefore, focused on the screening of the *aus* group, which is known for their adaptability to unfavorable environments. For example, the genotype FR13A harbors the submergence tolerance gene Sub1 (Xu et al., 2006), and Kasalath harbors the phosphorus starvation tolerance gene *Pstol1* (Gamuyao et al., 2012). In addition, DJ123 was shown to have high PUE (Wissuwa et al., 2015) and root efficiency (Mori et al., 2016).

Rice plant response to S deficiency started with leaf yellowing and visible reduction of shoot growth, followed by physiological and morphological modification of the roots, resulting in an increase in the root:shoot ratio, as deficiency became more severe. This S-deficiency response is related to adaptations within the S metabolic pathway first, and then related to the interaction with metabolic pathways of other nutrients (Lewandowska and Sirko, 2008; Kopriva et al., 2015).

In our experiment, S deficiency differentially induced root elongation and enhanced root growth in 30-day old plants (**Figure 2**). Similar responses have been reported for N, P and Magnesium (Mg) starvation (Hermans et al., 2006; Wissuwa et al., 2015), indicating that plants share a common mechanism to favor root growth to improve their ability to search and acquire scarce mineral nutrients from the soil. However, the two-way ANOVA revealed significant interaction effects on GxS (genotype by environment) for several traits including root length and root biomass, indicating that response to Sulfur treatment varies among the members of the aus group. Depending upon the breeding purpose, GxS interaction would be the very helpful to identify genotypes with good adaptation to specific S-deficiency conditions or with good ability to respond to broader nutritional problems.

We have further analyzed the trait association using two approaches: comparing the GWAS result for low S (**Figure 3**) and high S (**Supplementary Figure S3**-**1 in Presentation 1**) separately, and by calculating the association for the ratio low S/high S of phenotypic values. The GWAS comparison in two contrasting nutritional conditions (low S and high S) showed no common response. The identified QTLs did not co-localize indicating that specific and independent genetic effects were controlling the phenotypic response in both conditions (Rebolledo et al., 2016). On the other hand, GWAS result from relative values (low S/high S) revealed the presence of QTLs in greater number than in low-S treatment. Although the Manhattan plots showed many significant SNPs, which were rather scattered with undefined peak shape, there were several SNPs that co-localized in both analysis (**Supplementary Figure S3**-**2 in Presentation 1**), indicating that both approaches could be used complementarily.

The GWAS result indicated that the *aus* group possesses enough genetic variations for tolerance to S deficiency as several novel QTLs associated with agronomic traits, such as root length, and root and total dry matter were identified (**Table 3**). Among the favorable alleles in the significant loci there were two minor alleles for root dry matter and four for total dry matter.

Subsequently, based on the delineated LD blocks (**Supplementary Figure S5 in Presentation 1**), candidate genes with annotations putatively related to S metabolism and/or related processes to plant growth and stress response were selected for each locus.

A common response to S deficiency in most *aus* accessions was an increase in root length, and GWAS analysis identified one locus for which the common major haplotype increased root length, while only 17% of accessions with the minor haplotype had roots about 5% increase in length. Though these alleles appear to be the common alleles, this locus would be of little practical interest (from the agronomical/breeding point of view), candidate genes at this locus indicate the involvement in: a) regulation of growth in roots and flowers, and response to various stresses (helix-loop-helix transcription factor, Pires and Dolan, 2010; Hudson and Hudson, 2015); b) plant detoxification reaction during abiotic stress, like cadmium and arsenic detoxification (glutathione S-transferase, Kumar and Trivedi, 2018) and plant development (Gong et al., 2005); c) protein ubiquitination which plays roles in several plant developmental stages and several abiotic stress responses (ubiquitin-protein ligase enzymes; Sharma et al., 2016; Shu and Yang, 2017).

For root dry matter, main candidates belong to the family of a protein kinase (serine/threonine protein kinase) which is involved in the regulation of epidermal cell morphogenesis, root hair elongation, and possibly in plant growth and stress tolerance (Diédhiou et al., 2009).

For total dry matter, the sulfotransferase genes were selected as candidate genes because of their involvement in the secondary S-assimilation pathway. Sulfotransferase catalyzes the sulfation of several compounds to produce sulfate esters, sulfamates, and sulfate conjugates (Hirschman et al., 2014). In our study their expression pattern suggests that the one gene is differentially expressed in shoot tissue, but not induced by S-deficiency condition (**Figure 5**). Their transcript level abundance was consistently low, which agrees with the finding of Chen et al. (2012). The reported expression of Sulfotransferase genes suggests their involvement in plant defense, stress response, signaling and developmental regulation (Klein and Papenbrock, 2004; Hirschman et al., 2014), although their involvement in tolerance to sulfur tolerance is not yet understood. Further studies of gene expression at earlier stage of stress could contribute to elucidate their role.

The superior performance of a set of aus genotypes (Santhi Sufaid 207, Kangro, Juma and DJ123) harboring the positive haplotypes for root and total dry matter was confirmed in this study (**Figure 4**). Although, no difference among genotypes was found for total dry matter when relative values low S/high S was analyzed (**Supplementary Figure S4 in Presentation 1**), these genotypes with consistent superior performance would be agronomically desirable when selecting potential donors.

It is well known that S and N metabolism are highly integrated, for example, S deficiency can cause a reduction of synthesis of proteins, and accumulation of nitrogenous compounds, as well as lowering the utilization of available N in soil. Since the N level in low-input agrosystem could exacerbate the S deficiency problem, rice accessions/cultivars with S and N use efficiency should be considered in breeding program targeting yield and quality of rice.

To our knowledge, there are no reported QTLs for tolerance to S deficiency in rice, therefore this study represents a significant advance in current efforts to reduce S deficiency in agriculture. Furthermore, the rice accessions: Santhi Sufaid 207, Kangro, Juma and DJ123 could be used as donors to improve local cultivars (**Figure 4**). Although the *aus* group still has some negative agronomic traits, such as excessive plant height, shattering and lodging, the beneficial loci identified here can be transferred through conventional breeding and using the associated SNPs for marker assisted selection.

In summary, this study demonstrates the existence of genetic variability for tolerance to S-deficiency in the *aus* rice group. GWAS identified several QTLs associated with root and total dry matter where in the advantageous alleles were minor alleles, which could be exploited for improving modern rice cultivars, through marker assisted selection. In addition, the selected accessions, including DJ123 (which had already been identified as having high PUE and efficient P uptake), could be considered as donors in breeding for low-input conditions, in systems characterized by multiple nutrient deficiencies. This would allow for the development of new rice cultivars with tolerances to a range of nutrient deficiencies and overall improved nutrient-use efficiency. Thus, contributing towards efforts in achieving sustainable rice production and improved food security worldwide.

## Data Availability Statement

The SNP dataset used in this study (High Density Rice Array, HDRA) is available at the Rice diversity website: http://www.ricediversity.org/data. All phenotypic data produced in this study are included in the **Supplementary Material** (**Supplementary Table S5 in Data Sheet 2**).

## Author Contributions

JP-T, CB, and MW designed the experiments. CB and JP-T perform the experiments. JP-T and MW wrote the manuscript.

## Funding

This research was partially supported by the Science and Technology Research Partnership for Sustainable Development (SATREPS), Japan Science and Technology Agency (JST)/Japan International Cooperation Agency (JICA) Project (Grant No. JPMJSA1608).

## Conflict of Interest

The authors declare that the research was conducted in the absence of any commercial or financial relationships that could be construed as a potential conflict of interest.

## Supplementary Material

The Supplementary Material for this article can be found online at: https://www.frontiersin.org/articles/10.3389/fpls.2019.01668/full#supplementary-material
