# Supplementary material for: Identification of Loci Through Genome-Wide Association Studies to Improve Tolerance to Sulfur Deficiency in Rice
Source: Front Plant Sci. 2020 Jan 15;10:1668. doi: 10.3389/fpls.2019.01668 (PMC6975283; doi:10.3389/fpls.2019.01668)
Supplement: Supplementary file 1 [file Presentation_1.pptx]

## Slide 1
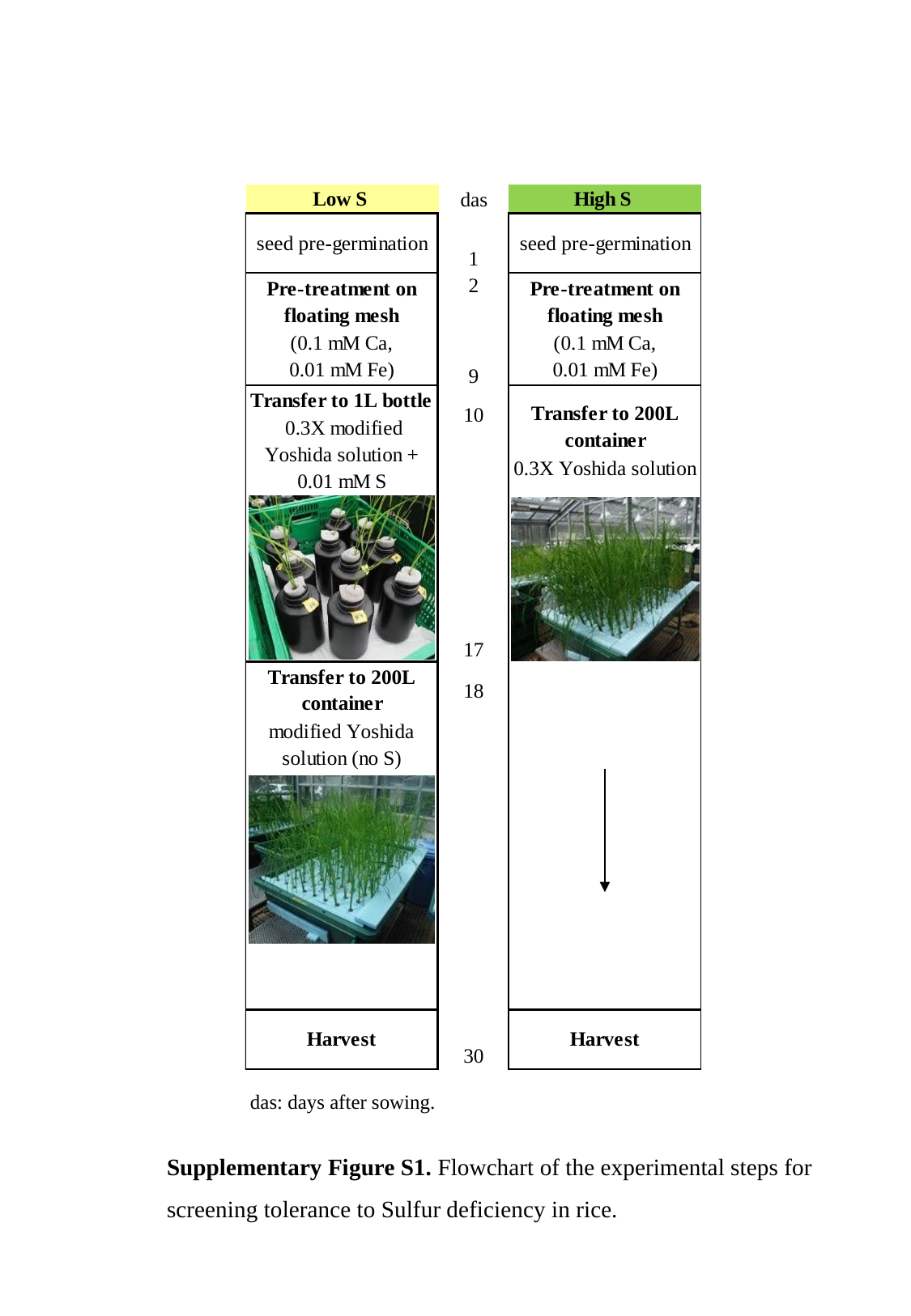

das: days after sowing.
Supplementary Figure S1. Flowchart of the experimental steps for screening tolerance to Sulfur deficiency in rice.

## Slide 2
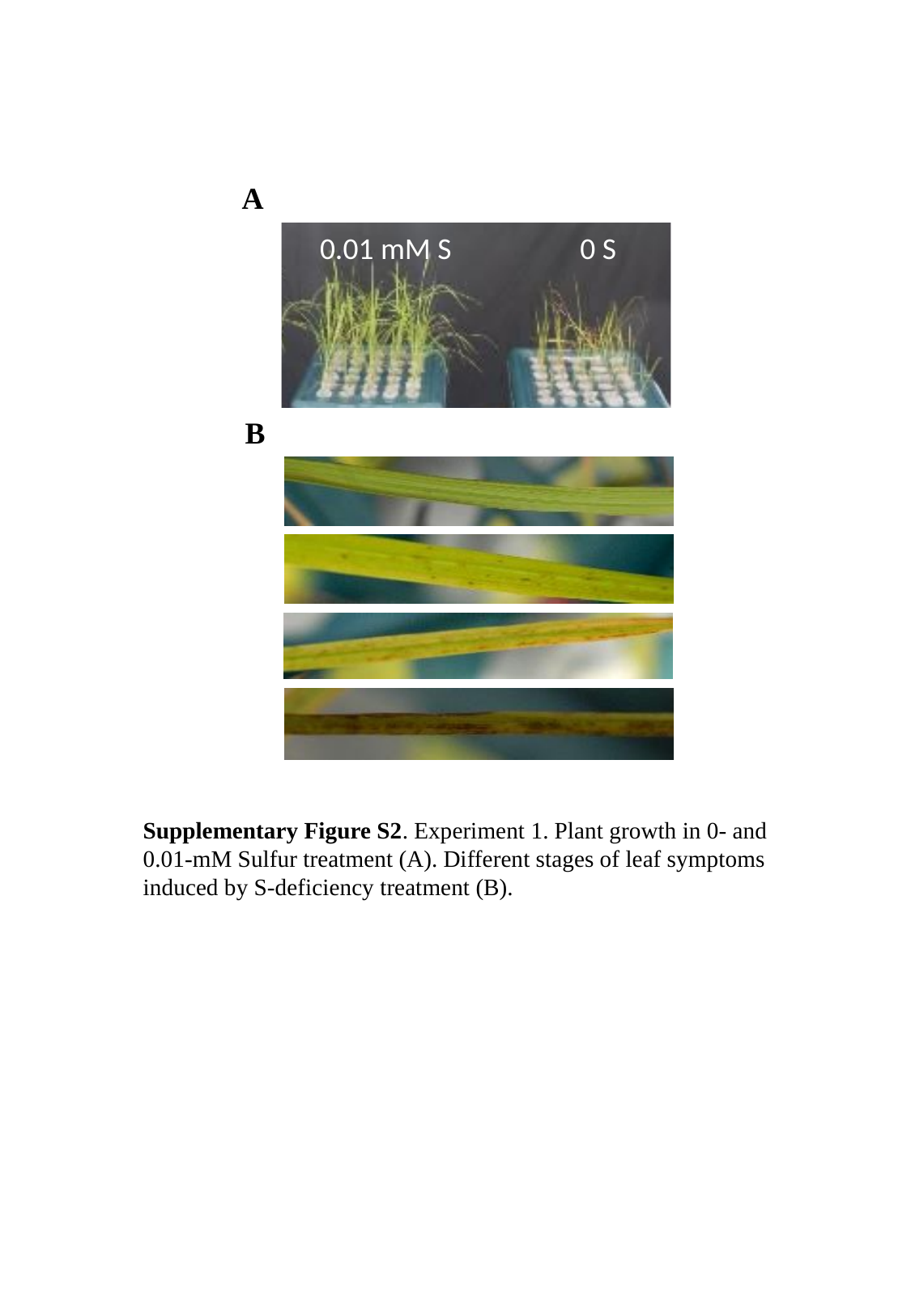

A
0.01 mM S
0 S
B
Supplementary Figure S2. Experiment 1. Plant growth in 0- and 0.01-mM Sulfur treatment (A). Different stages of leaf symptoms induced by S-deficiency treatment (B).

## Slide 3
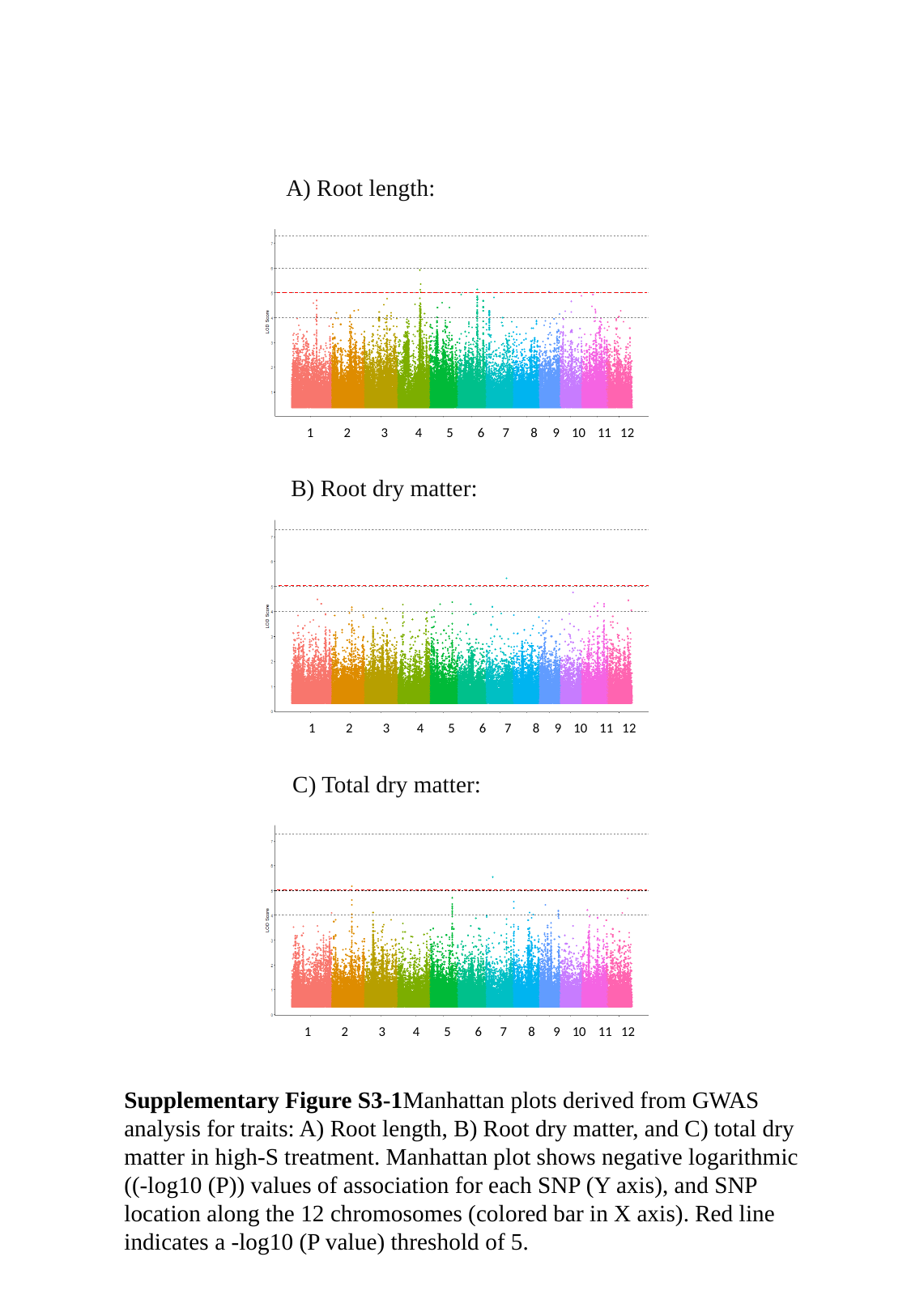

A) Root length:
 1 2 3 4 5 6 7 8 9 10 11 12
B) Root dry matter:
 1 2 3 4 5 6 7 8 9 10 11 12
C) Total dry matter:
 1 2 3 4 5 6 7 8 9 10 11 12
Supplementary Figure S3-1Manhattan plots derived from GWAS analysis for traits: A) Root length, B) Root dry matter, and C) total dry matter in high-S treatment. Manhattan plot shows negative logarithmic ((-log10 (P)) values of association for each SNP (Y axis), and SNP location along the 12 chromosomes (colored bar in X axis). Red line indicates a -log10 (P value) threshold of 5.

## Slide 4
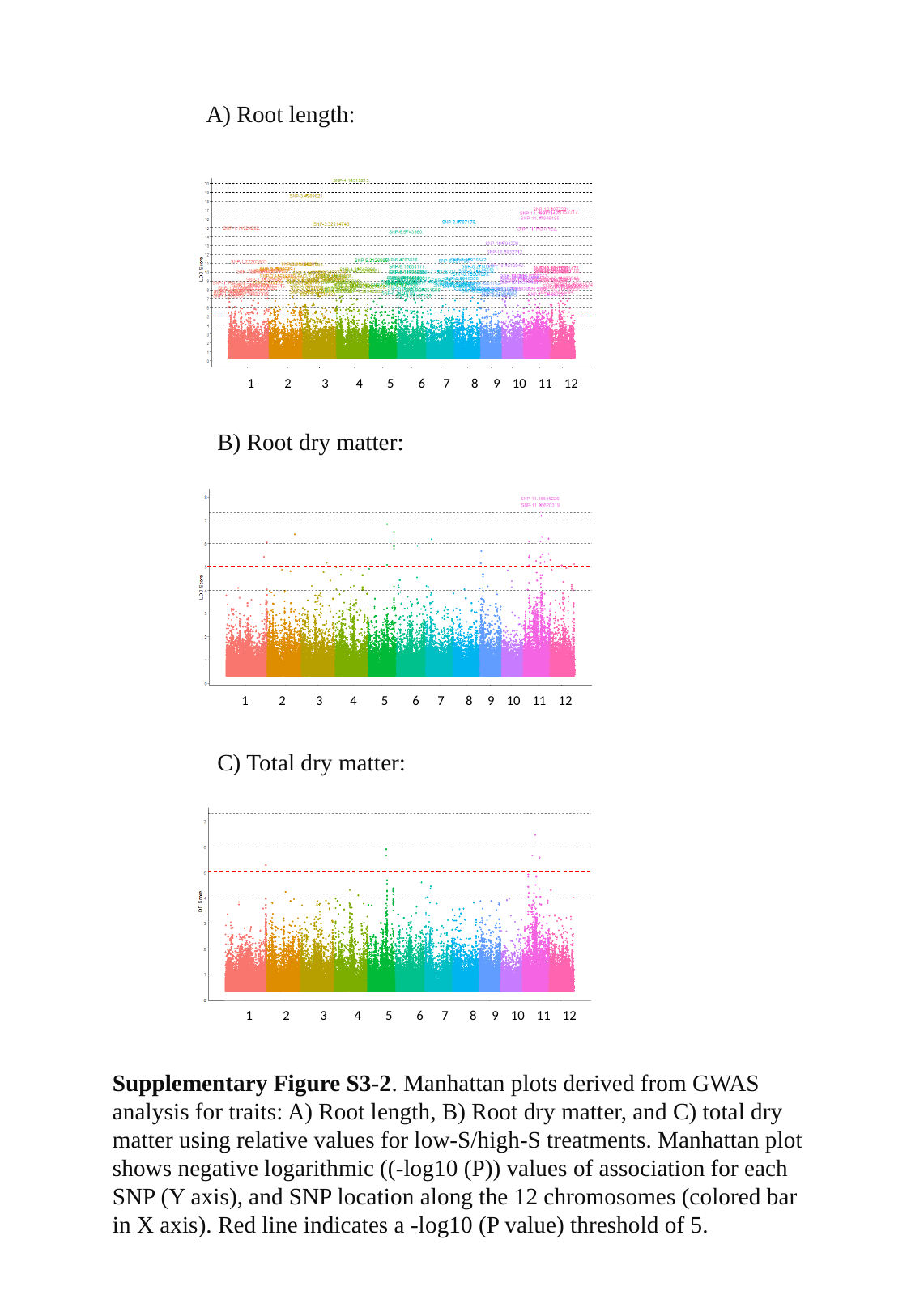

A) Root length:
 1 2 3 4 5 6 7 8 9 10 11 12
B) Root dry matter:
 1 2 3 4 5 6 7 8 9 10 11 12
C) Total dry matter:
 1 2 3 4 5 6 7 8 9 10 11 12
Supplementary Figure S3-2. Manhattan plots derived from GWAS analysis for traits: A) Root length, B) Root dry matter, and C) total dry matter using relative values for low-S/high-S treatments. Manhattan plot shows negative logarithmic ((-log10 (P)) values of association for each SNP (Y axis), and SNP location along the 12 chromosomes (colored bar in X axis). Red line indicates a -log10 (P value) threshold of 5.

## Slide 5
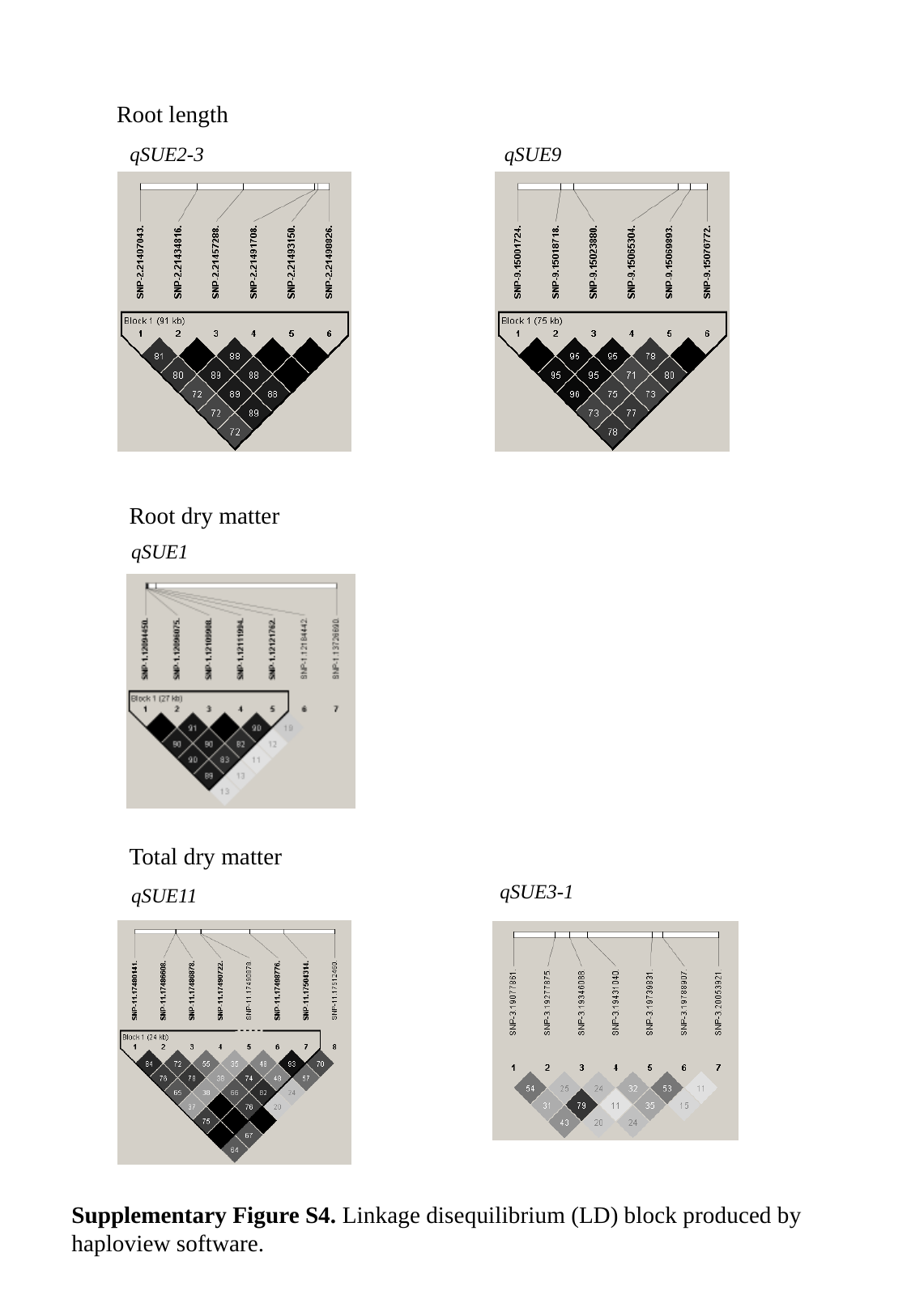

Root length
qSUE2-3
qSUE9
Root dry matter
qSUE1
Total dry matter
qSUE3-1
qSUE11
Supplementary Figure S4. Linkage disequilibrium (LD) block produced by haploview software.

## Slide 6
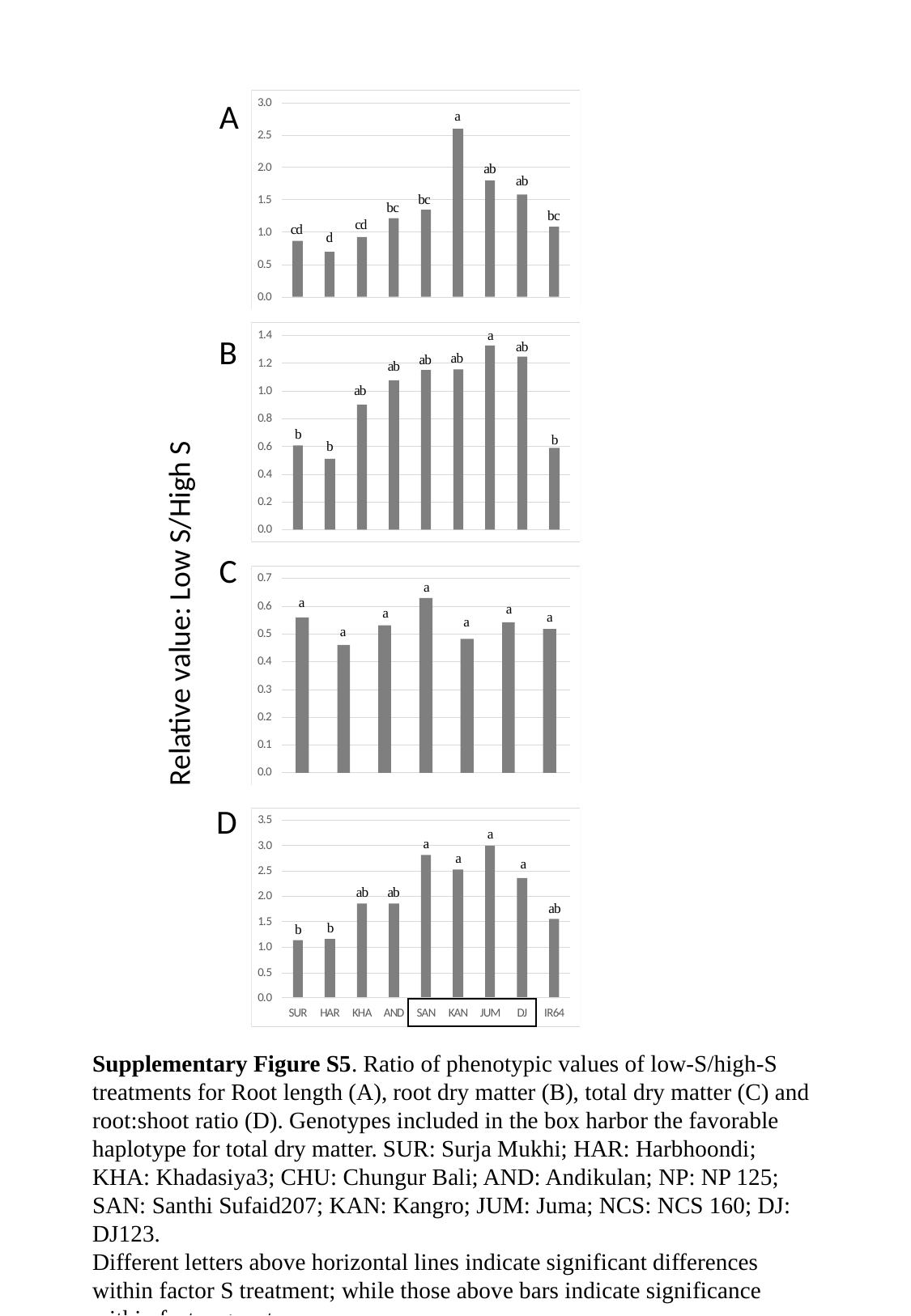

A
B
Relative value: Low S/High S
C
D
Supplementary Figure S5. Ratio of phenotypic values of low-S/high-S treatments for Root length (A), root dry matter (B), total dry matter (C) and root:shoot ratio (D). Genotypes included in the box harbor the favorable haplotype for total dry matter. SUR: Surja Mukhi; HAR: Harbhoondi; KHA: Khadasiya3; CHU: Chungur Bali; AND: Andikulan; NP: NP 125; SAN: Santhi Sufaid207; KAN: Kangro; JUM: Juma; NCS: NCS 160; DJ: DJ123.
Different letters above horizontal lines indicate significant differences within factor S treatment; while those above bars indicate significance within factor genotype.

## Slide 7
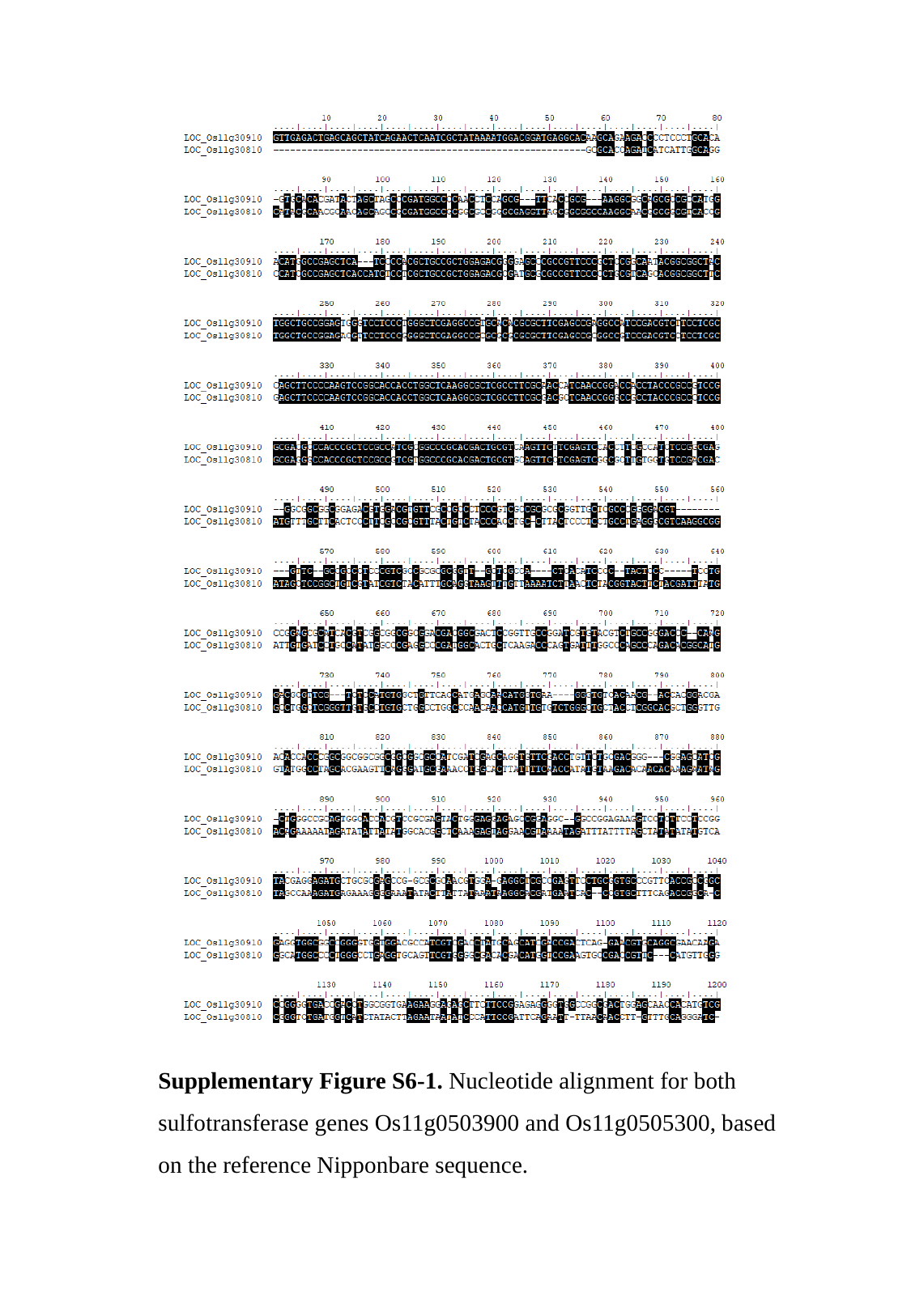

Supplementary Figure S6-1. Nucleotide alignment for both sulfotransferase genes Os11g0503900 and Os11g0505300, based on the reference Nipponbare sequence.

## Slide 8
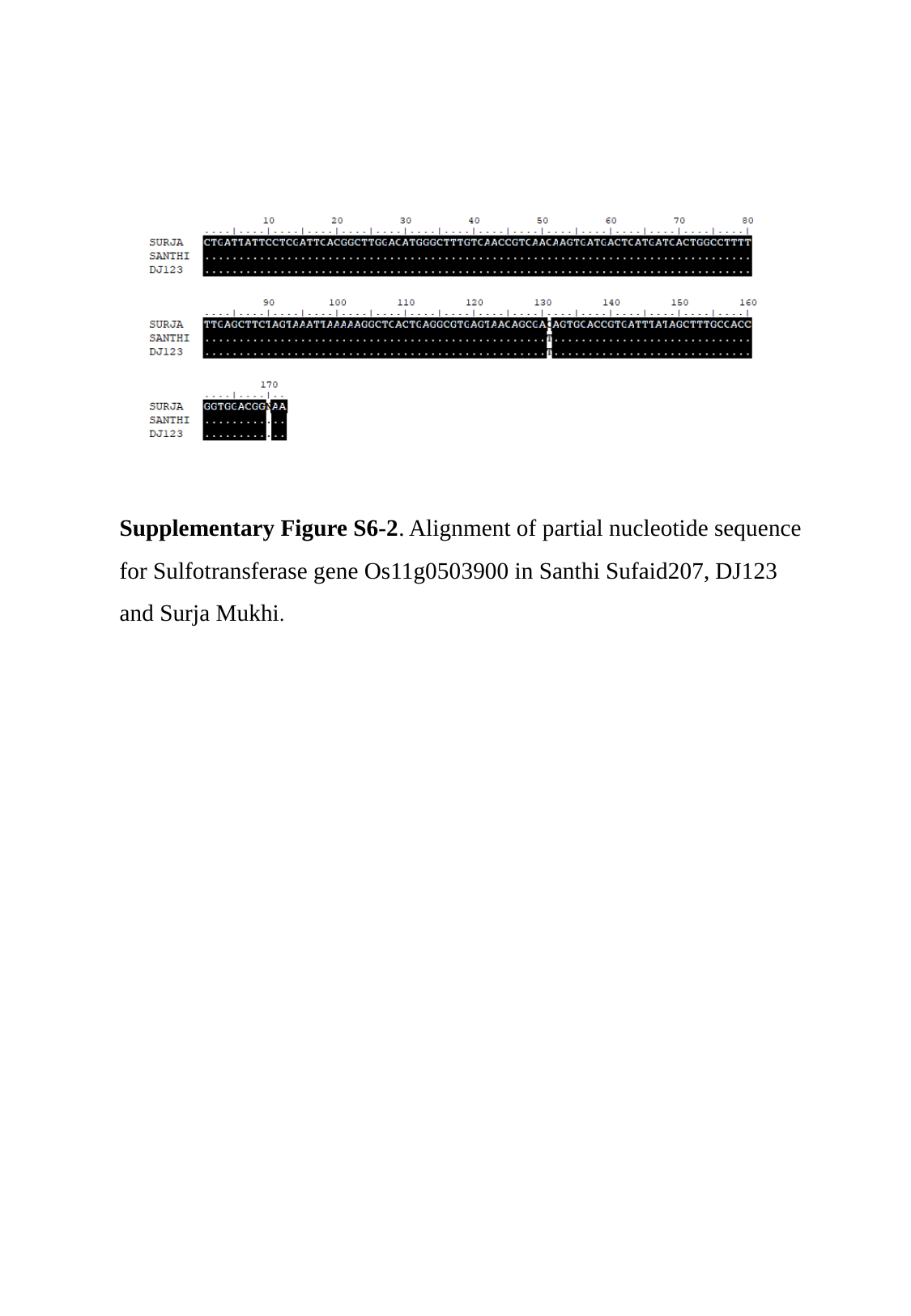

Supplementary Figure S6-2. Alignment of partial nucleotide sequence for Sulfotransferase gene Os11g0503900 in Santhi Sufaid207, DJ123 and Surja Mukhi.

## Slide 9
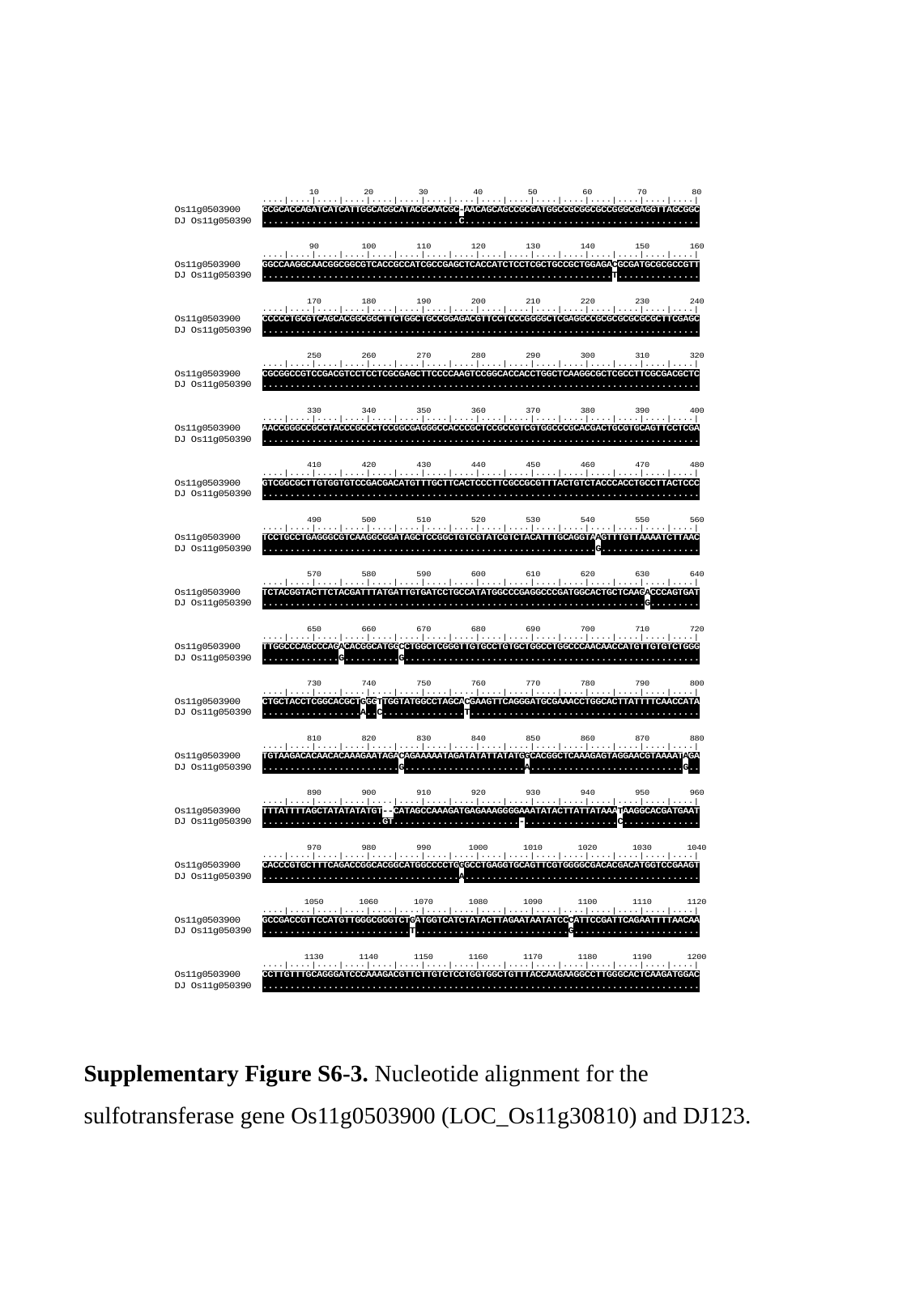

Supplementary Figure S6-3. Nucleotide alignment for the sulfotransferase gene Os11g0503900 (LOC_Os11g30810) and DJ123.

## Slide 10
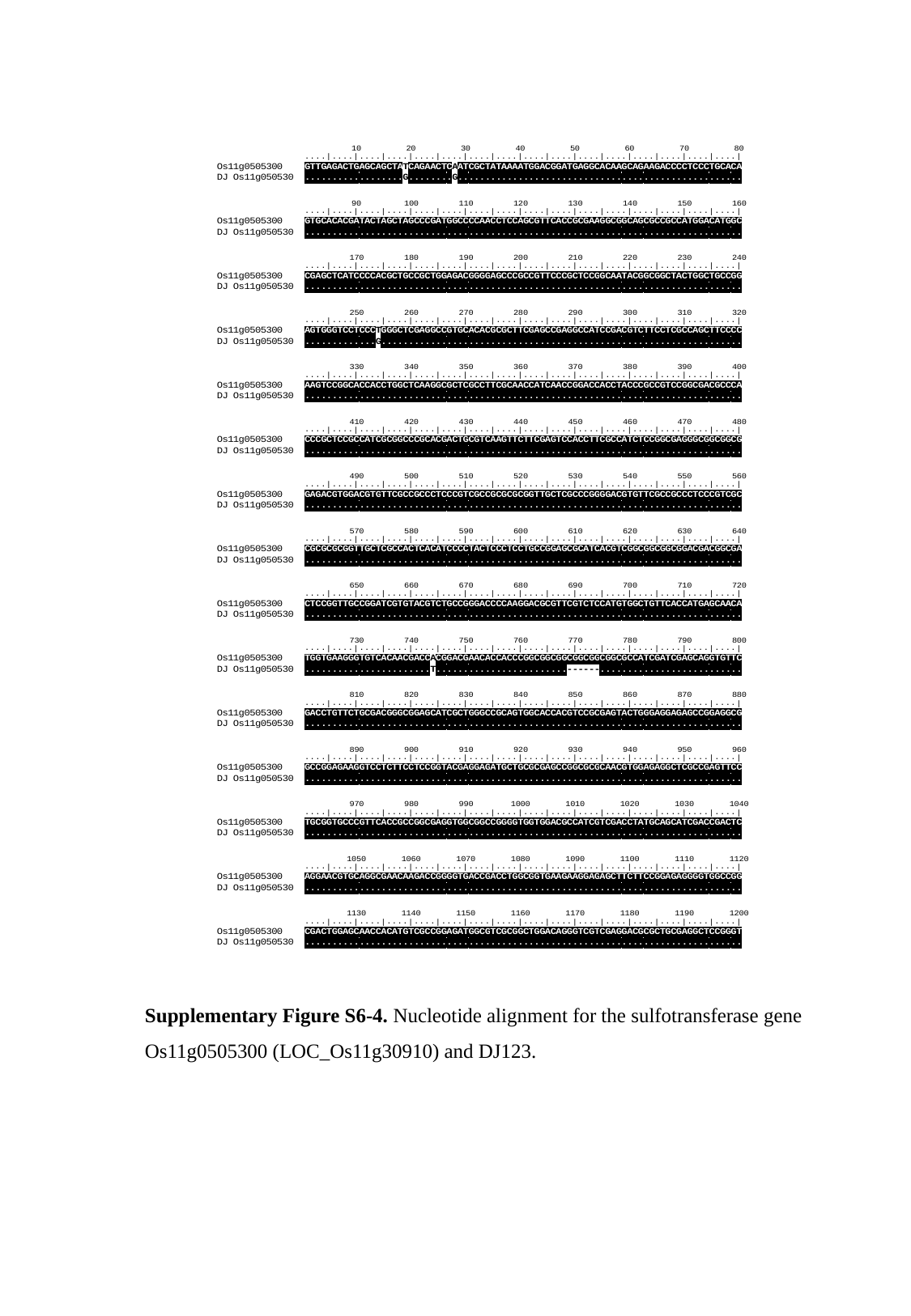

Supplementary Figure S6-4. Nucleotide alignment for the sulfotransferase gene Os11g0505300 (LOC_Os11g30910) and DJ123.
