# Supplementary material for: Identification of Loci Through Genome-Wide Association Studies to Improve Tolerance to Sulfur Deficiency in Rice
Source: Front Plant Sci. 2020 Jan 15;10:1668. doi: 10.3389/fpls.2019.01668 (PMC6975283; doi:10.3389/fpls.2019.01668)
Supplement: Supplementary file 2 [file DataSheet_1.docx]

**Supplementary Table S1**. Modified Yoshida culture solution for screening tolerance to S deficiency in rice plants.

**Supplementary Table S2**. Primers used in this study.

**Supplementary Table S3**. List of gene models included in GWAS associated loci

**Root length: *qSUE2-3***

***qSUE9***

**Root dry matter: *qSUE3-1***

**Root dry matter: *qSUE11***

**Supplementary Table S4**. Summary of ANOVA result for all experiments. Table S4-1 Experiment 1; Table S4-2 Experiment 3; Table S4-3 Gene expression result for Os11g0503900 and Os11g0505300.

**Table S4-1.** ANOVA result and descriptive statistics for experiment 1

ns: non-significant (P > 0.05), *: significant (P ≤ 0.05), **: (P ≤ 0.01), ***: (P ≤ 0.01).

LS: Low-S, HS; high-S treatment.

**Table S4-2.** ANOVA result and descriptive statistics for experiment 3

ns: non-significant (P > 0.05), *: significant (P ≤ 0.05), **: (P ≤ 0.01), ***: (P ≤ 0.01).

LS: Low-S, HS; high-S treatment.

**Table S4-3.** ANOVA result for gene expression of Os11g0503900 and Os11g0505300.

ns: non-significant (P > 0.05), *: significant (P ≤ 0.05), **: (P ≤ 0.01), ***: (P ≤ 0.01).

LS: Low-S, HS; high-S treatment.
